# An Investigation into the Relationship between Xanthan Gum Film Coating Materials and Predicted Oro-Esophageal Gliding Performance for Solid Oral Dosage Forms

**DOI:** 10.3390/pharmaceutics12121241

**Published:** 2020-12-20

**Authors:** Nélio Drumond, Sven Stegemann

**Affiliations:** Graz University of Technology, Inffeldgasse 13/III, 8010 Graz, Austria

**Keywords:** solid oral dosage forms, oro-esophageal transit, swallowing safety, film coating materials, xanthan gum, artificial mucous layer system, static friction, dynamic friction, predicted gliding performance, principal component analysis

## Abstract

Oral drug therapy is generally provided in the form of solid oral dosage forms (SODF) that have to be swallowed and move throughout the oro-esophageal system. Previous studies have provided evidence that the oro-esophageal transit of SODF depends on their shape, size, density, and surface characteristics. To estimate the impact of SODF surface coatings during esophageal transit, an in vitro system was implemented to investigate the gliding performance across an artificial mucous layer. In this work, formulations comprised of different slippery-inducing agents combined with a common film forming agent were evaluated using the artificial mucous layer system. Xanthan gum (XG) and polyethylene glycol 1500 (PEG) were applied as film-forming agents, while carnauba wax (CW), lecithin (LE), carrageenan (CA), gellan gum (GG) and sodium alginate (SA), and their combination with sodium lauryl sulfate (SLS), were applied as slippery-inducing components. All tested formulations presented lower static friction (SF) as compared to the negative control (uncoated disc, C, F0), whereas only CW/SLS-based formulations showed similar performance to F0 regarding dynamic friction (DF). The applied multivariate analysis approach allowed a higher level of detail to the evaluation and supported a better identification of excipients and respective concentrations that are predicted to improve in vivo swallowing safety.

## 1. Introduction

Solid oral dosage forms (SODF) are a major therapeutic intervention in healthcare provision due to their non-invasiveness and patient self-administration. There is growing concern related to safe swallowing by special populations such pediatrics, multimorbid and older patients due to SODF oro-gastric transport issues [1,2,3,4,5]. Problems in swallowing SODF have been shown to affect the effectiveness of drug therapies and to increase the incidence for inappropriate medicine alterations [6,7,8]. Furthermore, patients with impaired swallowing functions are more susceptible to medication-induced esophageal injuries caused by adhesion of the SODF to the esophageal mucosal tissue [9,10].

Previous studies have shown that the surface characteristics of SODF have a strong influence on adhesion and gliding properties when in contact with esophageal tissue [11,12]. To improve swallowability and increase safety during oral administration, investigations into coating surfaces of SODF that are non-mucosal adherent and slide easily throughout the esophagus along with the peristaltic movements are required [13].

Different in vitro systems have been proposed throughout the years to estimate the interaction of SODF polymer surface compositions with mucosal tissues. These methods were based on particle interaction or mechanical force evaluations [14,15,16,17], as well as on the measurement of the gliding resistance forces across artificial mucous layers or ex vivo animal-derived esophageal tissue [18,19,20]. To efficiently screen different polymer films, a simple artificial mucous layer system was developed to predict their resistance from initial contact with the mucous layer until later stages of gliding [21].

In this work, the artificial mucous layer system was used to characterize gum-based coating materials with regards to their in vitro gliding performance. Several formulations composed of xanthan gum (XG) and polyethylene glycol 1500 (PEG) as film forming agents, in combination with different slippery-inducing agents, were evaluated [13]. PEG was applied as a plasticizer material (1%) to support both the XG film formation process (coalescence) and overall gliding performance, taking into consideration previous work reported by the authors [21]. Furthermore, a multivariate analysis based on principal component analysis (PCA) was conducted to predict relationships between specific coating compositions and their oro-esophageal gliding performance.

## 2. Experimental Section

### 2.1. Materials

Polyethylene glycol MW 1500 (PEG), was purchased to Alfa Aesar (Lancashire, UK) and sodium alginate (SA) was obtained from Roth (Karlsruhe, Germany). Sodium lauryl sulfate (SLS) and lyophilized mucin from porcine stomach were purchased from Sigma-Aldrich (Munich, Germany). Gellan gum (GG) was supplied by CP Kelco (Atlanta, GA, USA). Lecithin (LE), carrageenan (CA) and xanthan gum (XG) were kindly provided by Cargill (Baupte, France). Finely powdered carnauba wax (CW) was a kind gift from the Freund Corporation (Tokyo, Japan) and double-sided adhesive carbon tape was purchased from Science Services (Munich, Germany). Gelatin strips (G) were donated by Capsugel (Colmar, France) and the water used was purified through a Milli-Q system (Merck Millipore, Darmstadt, Germany).

### 2.2. Preparation of Aqueous Coating Formulations

The coating compositions (Table 1) were prepared by mixing the appropriate amounts of formulation ingredients (using a magnetic stirrer) in purified water. The preparation was executed in three steps, with initial dispersion of the film forming polymers, followed by the addition of the remaining additives, and finished by adjusting the final weight of the formulation with water. Subsequently, the formulations were stirred at 300 RPM for 3 h until complete homogenization. For an easier identification, the tested formulations were combined based on their slippery-inducing agent: CW/SLS (F84–F86), LE/SLS (F87–F89), CA/SLS (F90–F92), GG/SLS (F93–F95) and SA/SLS (F96–F98).

### 2.3. Preparation of Film Coated Discs

The xanthan gum-based film coatings were produced from the aqueous polymer compositions using previously described solvent casting techniques (drying overnight in a vacuum oven at 50 °C) [22,23]. The thickness of the obtained polymer film coatings was approximately 200 ± 15 μm. Subsequently, the polymer films were precisely cut using a scalpel and fixed into the surface of the testing discs using universal double-sided adhesive tape (Figure S1). The surface area of the films was 7.065 cm^2^.

### 2.4. Evaluation of the Gliding Performance

**Figure 1:**
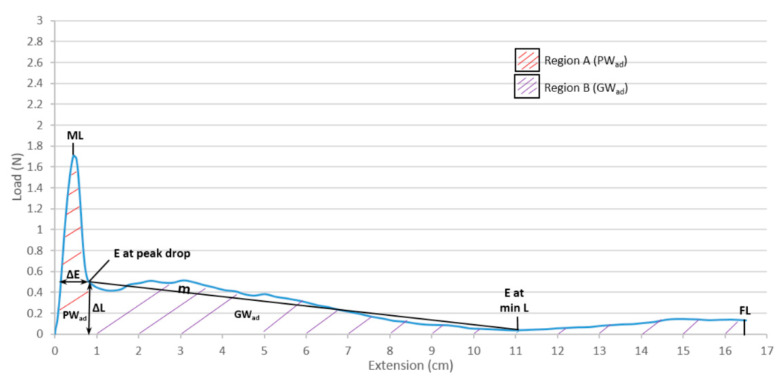
Curve interpretation for evaluation of the gliding performance: peak region A (PWad), gliding region B (GWad), maximum load (ML), maximum peak extension (ΔE), load at peak drop (ΔL), slope between E at peak drop to E at min load (m), final load (FL).

The in vitro apparatus consisted of the artificial mucous layer system described in an earlier publication [21]. All evaluations were performed at room temperature and fresh artificial mucous layers were prepared for every coated disc gliding assessment. After humidification of the mucous layer (Figure S2), the coated testing disc containing on top a 50 g weight was directed to the left end of the gliding region and the measurement was promptly initiated after contact of the coating with the mucous layer (no wetting time), in tension mode. The force (resistance) required to glide the coated disc at a constant speed of 2.6 cm/s through a gliding distance of 16.5 cm was measured with the load cell and automatically recorded with the equipment’s Bluehill^®^ software (Tarrytown, NY, USA). The relevant gliding parameters (Figure 1) were extrapolated from the gliding curves: maximum load (ML), peak work of adhesion (PW_ad_), gliding slope (*m*), load measured at final extension (FL), gliding work of adhesion (GW_ad_), and total work of adhesion (TW_ad_). Three replicates were performed for each polymer coating composition. G was applied as a positive control (F1) whereas the negative control (F0) was given by the uncoated disc (C).

### 2.5. Multivariate Analysis

Principal component analysis (PCA) was used to reduce the dimensionality of the different gliding variables into smaller datasets [24,25]. The purpose was to identify which concentrations and combinations of slippery-inducing agents could anticipate less resistance to movement. The outputs selected for the analysis were both static (SF) and dynamic frictions (DF), as these are important indicators for predictive gliding performance. The analysis was performed using Minitab^®^ 18 software (SquareCircle Global FZ LLC, Dubai, UAE).

## 3. Results

### 3.1. Gliding Performance Assessments

**Figure 2:**
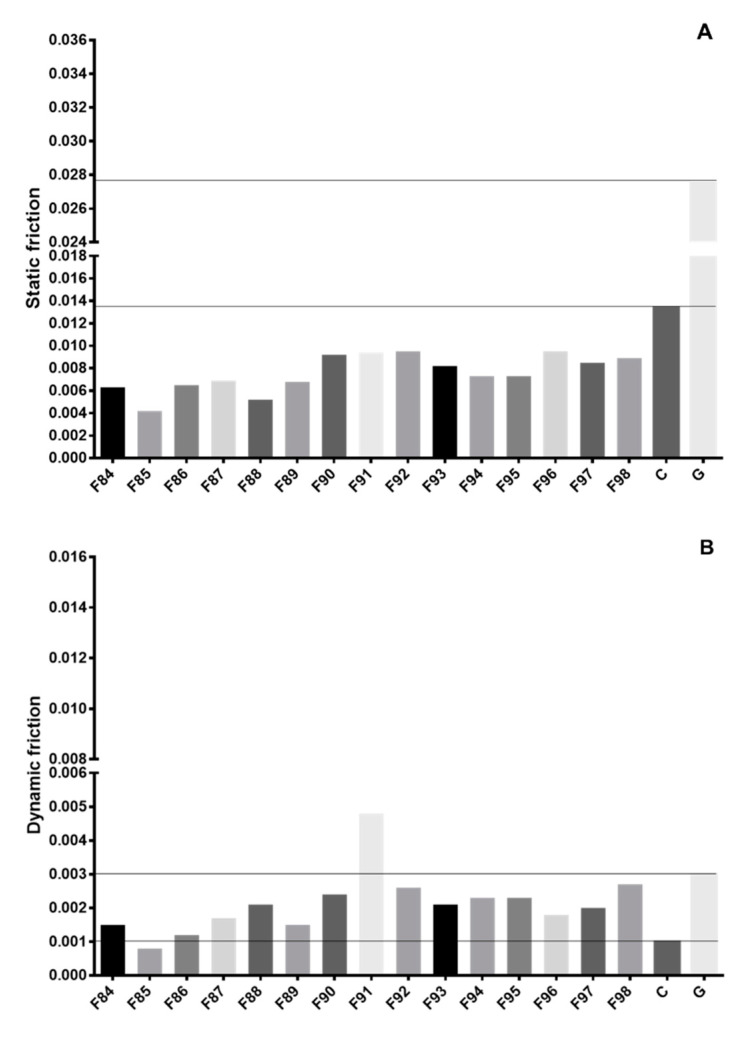
Static (SF) (**A**) and dynamic (DF) (**B**) friction obtained for xanthan gum/polyethylene glycol 1500 (XG/PEG)-based film coatings.

The gliding results obtained for the different formulations are summarized in Table 2. In addition, SF and DF were calculated for each formulation based on their gliding curves, and were applied in the multivariate analysis. As film-forming agents, XG/PEG showed an overall good performance with regards to the SF, as all formulations scored considerably lower when compared to C (Figure 2A). Formulations containing CW/SLS as slippery-inducing agents exhibited equivalent DF to C (Figure 2B), while the remaining formulations still showed lower DF when compared to G, with exception for formulation F91 (CA/SLS).

### 3.2. Multivariate Analysis

Only the first and the second components were applied to the PCA, as most of the data variation was represented within these two components.

#### 3.2.1. SF as Input for Predicted Gliding Performance

**Figure 3:**
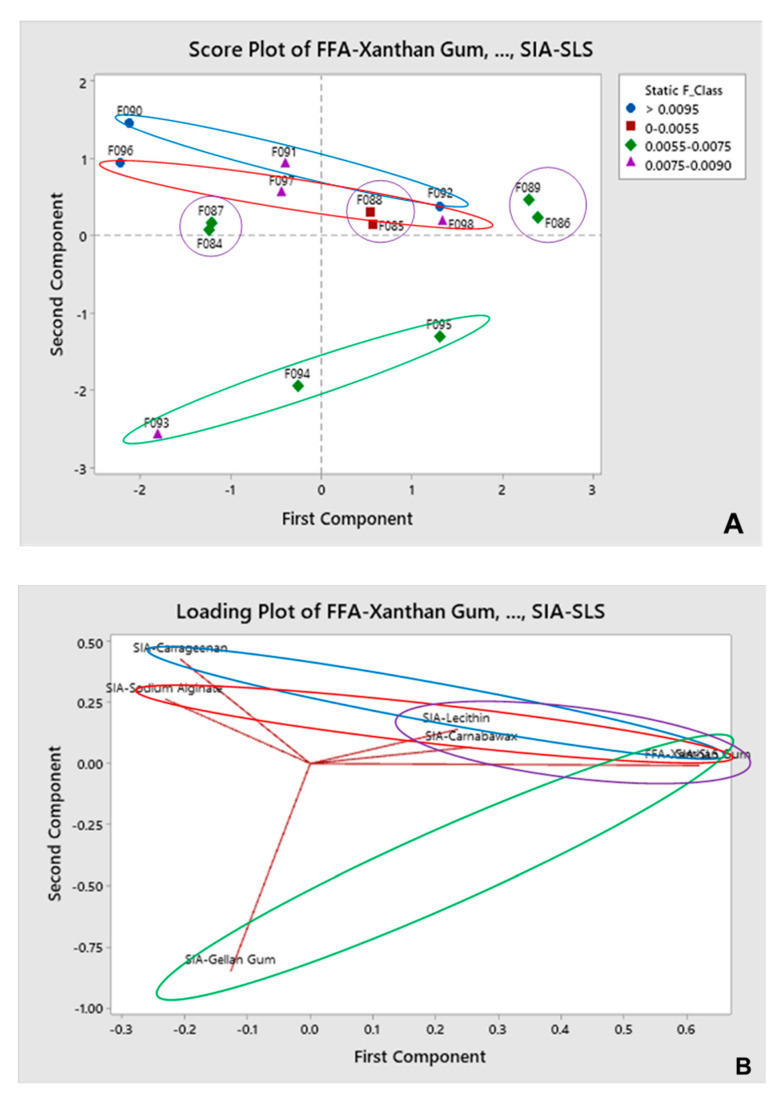
Score plot (**A**) and loading plot (**B**) obtained for XG/PEG-based coatings using SF as input for predicted gliding performance.

Intermediate concentrations for XG/PEG/CW/SLS and XG/PEG/LE/SLS (Figure 3) are suggested to present lower SF as compared to their upper and lower limits of concentration (purple). Combinations of XG/PEG/CA/SLS are predicted to present significant SF for all concentrations tested, whereas an intermediate concentration of XG appear to slightly reduce this parameter (blue). Films composed of XG/PEG/SA/SLS also exhibited significant SF for all concentrations tested, while a higher concentration of XG is expected to reduce the SF (red). The same prediction can be taken for films composed of XG/PEG/GG/SLS, as the increase in the concentration of XG reduced SF (green).

#### 3.2.2. DF as Input for Predicted Gliding Performance

**Figure 4:**
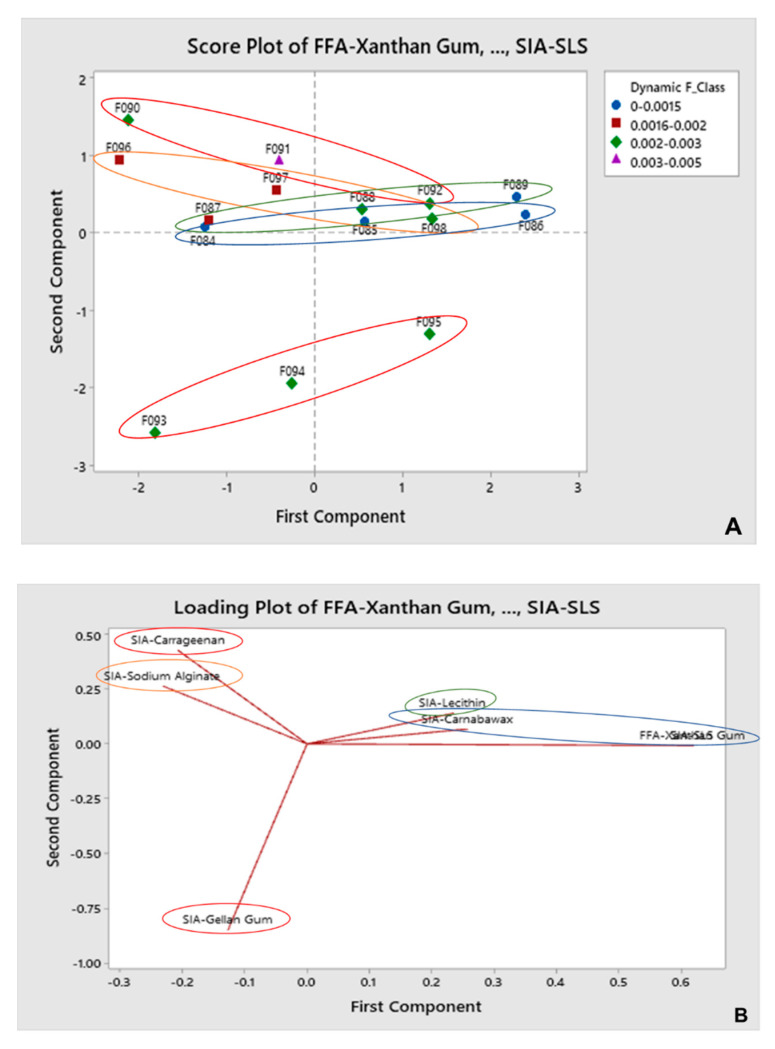
Score plot (**A**) and loading plot (**B**) obtained for XG/PEG-based coatings using DF as input for predicted gliding performance.

Combinations of XG/PEG/CA/SLS and XG/PEG/GG/SLS suggest a considerable degree of DF (Figure 4) with regard to the tested concentrations (red). Higher concentrations for XG/PEG/LE/SLS films are predicted to decrease DF (green). Increasing concentration of XG/PEG/SA/SLS predict higher DF, and as such, low and medium concentrations should anticipate less resistance to movement (orange). Overall, a lower DF is predicted for films composed of XG/PEG/CW/SLS, with all tested concentrations anticipating enhanced gliding performance as compared to other film coating compositions (blue).

## 4. Discussion

The artificial mucous layer system used in this study enables a basic characterization of the gliding performance of different film coating materials and can be applied for the screening and formulation design of patient-centric coatings to ensure SODF swallowing safety in special patient populations.

The collected gliding curves are characterized by two main domains: a starting peak related to the force required to overcome the initial SF, and a second region (after peak drop) representing the DF (kinetic) of the coating material across the artificial mucous layer (Figure 1). Both SF and DF are very important for quantifying the overall gliding performance, especially when compared to F0 and F1, and as such were analyzed in more detail through the PCA. The analysis allowed a “spatial distribution” of the datasets in the score plots, which was then complemented by the loading plots showing what specific formulations are driving (and in which magnitude) the dataset with regard to the desired output parameter. Therefore, the PCA is a useful instrument in combination with the artificial mucous layer system, as it allows us to correlate the impact of different variables in order to achieve the desired output. In addition, the level of detail obtained from the analysis supported a better identification of which combination of excipients, including their specific concentrations, contribute to film coating materials that are predicted to generate lower SF and DF profiles across mucosal surfaces.

PEG is typically applied to coating formulations as plasticizer materials (in concentrations between 1.0–1.5%) to support film coalescence [13]. In addition, due to their waxy nature, PEG grades were shown to exhibit enhanced gliding properties in previous works (Figure S3), with a loss of performance (PEG 1500 < 3350 < 6000) being correlated to increasing molecular weight [21]. In addition, PEG 1500 was applied in a concentration of 1% to all film coating formulations investigated in this work, as complementary assessments have shown that 0.5% increments in the concentration of PEG (up to 3.0%) do not really reflect an improvement in the gliding performance of manufactured films, with higher dynamic frictions being respectively measured (data not shown). The different slippery-inducing agents applied in the coating formulations promoted lower SF (Figure 2A) when compared to the uncoated disc (C. F0) and the gelatin strip (G, F1), indicating that the tested film materials present lower resistance to start gliding the disc in the artificial mucous layer. The scenario was slightly different with regard to the DF, as CW/SLS was the only slippery-inducing agent to show similar performance to F0. Yet, all remaining formulations still demonstrated lower DF in relation to F1 (with the exception of F91), which is predictive of lower mucoadhesive potential at later stages of gliding. As such, CW/SLS are seen as optimal slippery-inducing agents to be combined with XG/PEG film coating compositions, whereas CA and GG should be avoided. Depending on the concentration level, LE/SLS and SA/SLS may also be applied, but their predictive potential is still expected to be lower than CW/SLS.

Previous works have already demonstrated the benefits of using XG as a film-coating material to improve the easiness of swallowing valsartan tablets [26]. In addition, combinations of SA/SLS have been demonstrated to enhance the gliding performance of manufactured films [27], while the usability of alginates in the development of tablets to improve swallowability and medication administration has also been reported in the literature [28].

The formulations tested on the artificial mucous layer system performed distinctively with regard to their measured SF and DF, which demonstrates that the method is valid for characterizing the gliding behavior of different coating materials. Furthermore, the method also allows one to identify proper slippery-inducing agents that should be combined with given film forming agents (XG/PEG in this work), to design patient-centric coating materials that are predicted to promote swallowing safety during the administration of SODF by special patient populations. Therefore, the data generated in this work may be helpful to provide guidance in the future to pharmaceutical technology researchers when formulating easy-to-swallow coating materials [29,30].

While this study provides relevant comparative data on the predicted gliding performance for different coating formulations, further investigations should be conducted to assess their in vivo performance when applied to SODF, by tracking the course and/or velocity of the coated SODF throughout the patients’ oro-esophageal system using validated methodology such as real-time magnetic marker monitoring [31] or video fluoroscopy [32]. Future work should include further characterization of the shortlisted formulations with regard to their functional groups (to confirm miscibility) and surface roughness via FTIR spectroscopy and SEM analysis, respectively, especially when applying the coating formulations using different coating technologies and tablets sizes/shapes. Lastly, it should be noted that the method applied presents some limitations, as the conditions of the in vivo method might not represent truly biorelevant conditions (e.g., temperature). These deviations were considered acceptable since the investigation was a comparative study of the slip properties of different films.

## 5. Conclusions

The artificial mucous layer system was applied in this work to evaluate the gliding performance of coating formulations designed with different combinations of slippery-inducing agents to common film forming agents (xanthan gum/PEG). A PCA was performed to evaluate the gliding profiles and predict which combinations of excipients, including their specific concentrations, are desired to reduce SF and DF. Film coating materials composed of XG/PEG/CW/SLS demonstrated enhanced gliding characteristics compared to C and G. The measurements allowed us to differentiate gliding performance between different combinations and may support predictions for in vivo swallowability, which need to be confirmed in further trials. Lastly, strategies to be adopted when formulating film coating materials intended to display enhanced gliding performance are also suggested.

## Figures and Tables

**Table 1:** Composition of manufactured film-coating materials.

Coating	Film-Forming Agents	Slippery-Inducing Agents
F84–F86	XG 0.40–0.60%, PEG 1.0%	CW 0.10–0.5%, SLS 0.20–0.60%
F87–F89	XG 0.40–0.60%, PEG 1.0%	LE 0.30–1.10%, SLS 0.20–0.60%
F90–F92	XG 0.40–0.60%, PEG 1.0%	CA 0.30–1.10%, SLS 0.20–0.60%
F93–F95	XG 0.40–0.60%, PEG 1.0%	GG 0.10–0.20%, SLS 0.20–0.60%
F96–F98	XG 0.40–0.60%, PEG 1.0%	SA 0.10–0.50%, SLS 0.20–0.60%

**Table 2:** Gliding performance parameters obtained for the film coatings tested *.

Coating	ML (N)	PWad (mJ)	*m*	FL (N)	Gwad (mJ)	TWad (mJ)	SF	DF
F84	0.39 ± 0.10	1.94 ± 0.14	0.01 ± 0.00	0.05 ± 0.00	14.12 ± 2.65	16.06 ± 2.69	0.0063	0.0015
F85	0.26 ± 0.09	0.94 ± 0.03	0.00 ± 0.00	0.05 ± 0.00	7.43 ± 1.05	8.37 ± 0.54	0.0042	0.0008
F86	0.40 ± 0.02	2.37 ± 0.55	0.00 ± 0.00	0.05 ± 0.00	11.08 ± 0.99	13.45 ± 0.99	0.0065	0.0012
F87	0.43 ± 0.04	2.99 ± 0.47	0.01 ± 0.00	0.04 ± 0.00	16.14 ± 0.84	19.13 ± 1.00	0.0069	0.0017
F88	0.32 ± 0.02	1.65 ± 0.22	0.01 ± 0.00	0.08 ± 0.00	21.04 ± 3.65	22.69 ± 1.47	0.0052	0.0021
F89	0.42 ± 0.03	3.21 ± 0.36	0.01 ± 0.00	0.05 ± 0.01	14.66 ± 2.54	17.69 ± 1.02	0.0068	0.0015
F90	0.67 ± 0.09	5.18 ± 1.21	0.02 ± 0.01	0.05 ± 0.00	22.91 ± 4.65	28.09 ± 2.36	0.0092	0.0024
F91	0.58 ± 0.01	5.06 ± 1.33	0.01 ± 0.00	0.20 ± 0.00	37.66 ± 3.65	42.79 ± 8.47	0.0094	0.0048
F92	0.59 ± 0.05	3.64 ± 0.55	0.01 ± 0.00	0.15 ± 0.00	24.94 ± 2.22	28.58 ± 4.65	0.0095	0.0026
F93	0.51 ± 0.03	3.17 ± 0.74	0.01 ± 0.00	0.10 ± 0.00	19.09 ± 4.56	22.26 ± 6.87	0.0082	0.0021
F94	0.45 ± 0.02	2.47 ± 0.88	0.00 ± 0.00	0.11 ± 0.00	21.84 ± 1.85	24.31 ± 4.54	0.0073	0.0023
F95	0.45 ± 0.01	3.74 ± 0.33	0.00 ± 0.00	0.13 ± 0.00	20.81 ± 3.65	24.55 ± 4.52	0.0073	0.0023
F96	0.59 ± 0.06	6.53 ± 0.21	0.01 ± 0.00	0.06 ± 0.00	16.16 ± 0.58	22.69 ± 3.99	0.0095	0.0018
F97	0.53 ± 0.05	4.59 ± 0.11	0.02 ± 0.00	0.06 ± 0.00	18.31 ± 0.97	22.91 ± 3.54	0.0085	0.0020
F98	0.55 ± 0.04	4.84 ± 0.14	0.02 ± 0.01	0.11 ± 0.01	25.87 ± 2.54	30.70 ± 4.44	0.0089	0.0027
F1	1.71 ± 0.15	8.34 ± 1.36	0.03 ± 0.10	0.07 ± 0.01	25.43 ± 4.25	37.99 ± 1.58	0.0276	0.0030
F0	0.83 ± 0.12	4.02 ± 0.58	0.02 ± 0.00	0.00 ± 0.00	7.35 ± 1.36	11.37 ± 0.25	0.0134	0.0010

* Average results ± SD of three measurements (*n* = 3). SD for SF and DF not given (approx. zero).

## Supplementary Materials

The following are available online at https://www.mdpi.com/1999-4923/12/12/1241/s1, Figure S1. Preparation of coating materials via film casting technique (drying in oven). Figure S2. Schematic illustration of the steps involved in the preparation of the artificial mucous layer: (A) frame with PTFE-coated sliding base, (B) distribution of the mucin powder in the frame, (C) compression of the mucin power with a PTFE-coated press, (D) press withdrawal and retention of the homogeneous mucous layer in the lower base of the frame, (E) base sliding with distribution of the mucin surface in the carbon adhesive tape, (F) moistening of the artificial mucous layer by spraying water to six specific central positions in the gliding region. Figure S3. Overall enhanced gliding performance obtained for PEG grades in previous developed work by the authors (Drumond and Stegemann, 2019): (A) negative correlation observed between increasing molecular weight of PEG grade and gliding performance; (B) optimal gliding performance obtained for PEG 1500 as compared to other tested film coatings.
