# Supplementary material for: An Investigation into the Relationship between Xanthan Gum Film Coating Materials and Predicted Oro-Esophageal Gliding Performance for Solid Oral Dosage Forms
Source: Pharmaceutics. 2020 Dec 20;12(12):1241. doi: 10.3390/pharmaceutics12121241 (PMC7765896; doi:10.3390/pharmaceutics12121241)
Supplement: Supplementary file 1 [file pharmaceutics-12-01241-s001.pdf]

pharmaceutics

Supplementary Materials: An Investigation into the Relationship between Xanthan Gum Film Coating Materials and Predicted Oro-Esophageal Gliding Performance for Solid Oral Dosage Forms

Nélio Drumond and Sven Stegemann

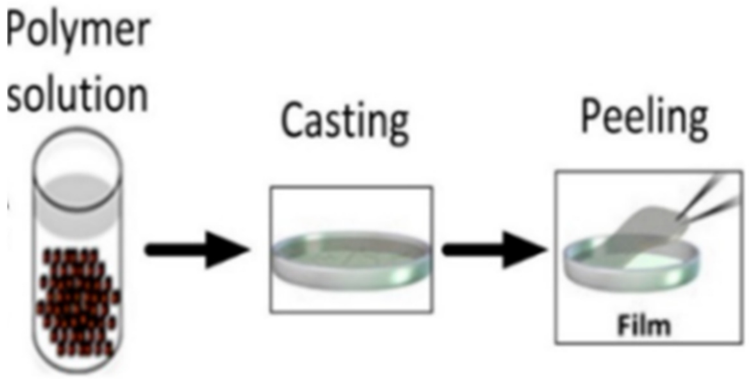

Figure S1. Preparation of coating materials via film casting technique (drying in oven).

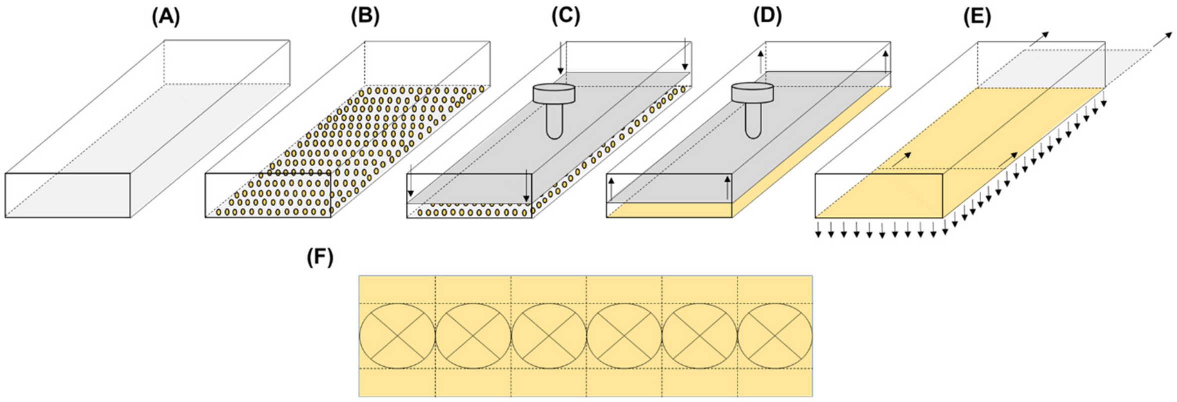

Figure S2. Schematic illustration of the steps involved in the preparation of the artificial mucous layer: (**A**) frame with PTFE-coated sliding base, (**B**) distribution of the mucin powder in the frame, (**C**) compression of the mucin power with a PTFE-coated press, (**D**) press withdrawal and retention of the homogeneous mucous layer in the lower base of the frame, (**E**) base sliding with distribution of the mucin surface in the carbon adhesive tape, (**F**) moistening of the artificial mucous layer by spraying water to six specific central positions in the gliding region.

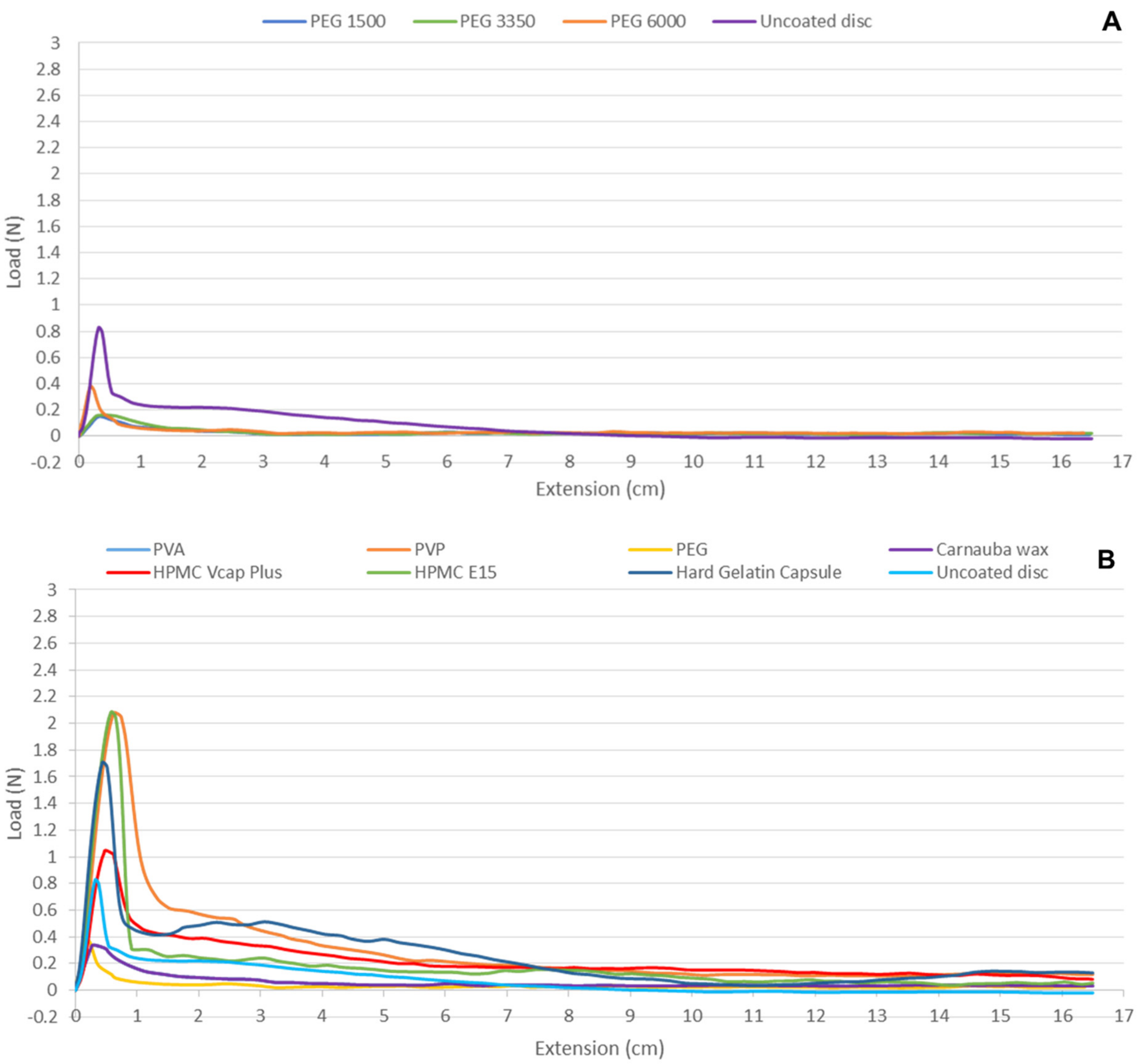

Figure S3. Overall enhanced gliding performance obtained for PEG grades in previous developed work by the authors (Drumond and Stegemann, 2019): (**A**) negative correlation observed between increasing molecular weight of PEG grade and gliding performance; (**B**) optimal gliding performance obtained for PEG 1500 as compared to other tested film coatings.
